# The comparison of gut gene expression and bacterial community in *Diaphorina citri* (Hemiptera: Liviidae) adults fed on *Murraya exotica* and ‘Shatangju’ mandarin (*Citrus reticulate* cv. Shatangju)

**DOI:** 10.1186/s12864-023-09308-2

**Published:** 2023-07-24

**Authors:** Jinghua Dai, Xueming Cai, Luyang Liu, Yanzheng Lin, Yuting Huang, Jintian Lin, Benshui Shu

**Affiliations:** Guangzhou City Key Laboratory of Subtropical Fruit Trees Outbreak Control, Institute for Management of Invasive Alien Species, Zhongkai University of Agriculture and Engineering, 313 Yingdong Teaching Building, Guangzhou, 510225 China

**Keywords:** *Diaphorina citri*, Host plants, RNA-seq, Differentially expressed genes, 16S rDNA, Gut bacterial community

## Abstract

**Background:**

*Diaphorina citri* Kuwayama is an important citrus pest. It serves as the vector for the transmission of *Candidatus* Liberibacter asiaticus (*C*Las), which induced a destructive disease, Huanglongbing, and caused huge economic losses. During the interaction between insects and plants, insects have evolved a series of mechanisms to adapt to various host plants. *Murraya exotica* and ‘Shatangju’ mandarin (*Citrus reticulate* cv. Shatangju) are the Rutaceae species from different genera that have been discovered as suitable hosts for *D. citri* adults. While the adaptation mechanism of this pest to these two host plants is unclear.

**Results:**

In this study, RNA-seq and 16 S rDNA amplification sequencing were performed on the gut of *D. citri* adults reared on *M. exotica* and ‘Shatangju’ mandarin. RNA-seq results showed that a total of 964 differentially expressed genes were found in different gut groups with two host plant treatments. The impacted genes include those that encode ribosomal proteins, cathepsins, and mitochondrial respiratory chain complexes. According to 16 S rDNA sequencing, the compositions of the gut bacterial communities were altered by different treatments. The α and β diversity analyses confirmed that the host plant changes influenced the gut microbial diversity. The functional classification analysis by Tax4Fun revealed that 27 KEGG pathways, mostly those related to metabolism, including those for nucleotide metabolism, energy metabolism, metabolism of cofactors and vitamins, amino acid metabolism, carbohydrate metabolism, xenbiotics biodegradation and metabolism, lipid metabolism, and biosynthesis of other secondary metabolites, were significantly altered.

**Conclusion:**

Our preliminary findings shed light on the connection between *D. citri* and host plants by showing that host plants alter the gene expression profiles and bacterial community composition of *D. citri* adults.

**Supplementary Information:**

The online version contains supplementary material available at 10.1186/s12864-023-09308-2.

## Background

The Asian citrus psyllid (ACP), *Diaphorina citri* Kuwayama (Hemiptera: Liviidae), is one of the most serious citrus pests worldwide due to its role as a vector of the uncultured proteobacterium *Candidatus* Liberibacter asiaticus (*C*Las) that causes Huanglongbing (HLB) [1, 2]. As a phytophagous pest, *D. citri* feeds on the phloem sap of plants and has a narrow host range in the family Rutaceae, such as the commercial citrus species *Citrus limon*, *C. sinensis*, *C. aurantium*, *C. paradisi*, *C. aurantifolia*, *C. maxima*, and *C. reticulata* Blanco, as well as other economic plants *Clausena lansium* and *Murraya exotica* [3–5]. The performance of *D. citri* feeding on different host plants was different. For instance, the developmental period and survival rate of *D. citri* were different when feeding on *C. paradisi*, *C. aurantium*, *C. aurantifolia*, and *C. sinensis*, respectively [6]. In addition, the life-table parameters and host preference of *D. citri* on *C. reticulata* cv. Shatangju, *C. reticulata* cv. Ponkan, *M. exotica*, *C. limon*, and *C. sinensis* have been evaluated [5]. Furthermore, the different fitness of *D. citri* fed on *C. sinensis* and *M. exotica* was also investigated [7].

RNA-seq is a conventional technical approach for studying insect plasticity triggered by different host plants at the transcriptional level [8]. The transcriptional plasticity of some insects in adaptation to different host plants was investigated, including *Sitobion avenae* and *Monochamus saltuarius* [9, 10]. As a highly regenerative tissue, the multi-functional gut in adult insects can renew itself rapidly in response to shifting inputs from internal and external stimuli, including various nutrients ingested, toxic substances, and endogenous signaling molecules [11]. In insect-plant interactions, the insects were able to quickly change their gut physiology to accommodate host plants with different chemical compositions, including plant-derived nutrition and defensive chemicals [12]. The genome-wide midgut transcriptome analysis found that the lepidopteran herbivore *Trichoplusia ni* adjusts its gut physiology by altering the mRNA expression of genes encoding digestive enzymes and detoxifying enzymes to deal with diverse host plants [13].

Insect guts are also important physiological tissues and are favorable for the colonization of microorganisms because of their attributes, such as simple access to nutrients and defense against diverse environmental stressors [14]. The gut microbes are indispensable because they offer benefits to the host in multiple aspects, including detoxification of plant allelochemicals, provision of nutrition for host insects, and digestion of complex carbohydrates [15, 16]. Intestinal microorganisms are engaged in several life processes of host insects, such as maintaining intestinal homeostasis, increasing oviposition, fecundity, and longevity of the host, controlling the insect immune system to protect against injury from external organisms, and altering insect behavior [17–19]. The microorganisms of *D. citri* have also recently received widespread attention. Five different species of endosymbiotic microbiota of *D. citri*, including the mycetocyte symbiont, the syncytium symbiont, *Arsenophonus* sp., *Wolbachia* sp., and Liberobacter sp., were discovered [20]. The microbial diversity of *D. citri* was also studied by restriction fragment length polymorphism (RFLP) and denaturing gradient gel electrophoresis (DGGE) analyses of 16 S rDNA sequences, and the results showed that the syncytium endosymbiont is a dominant bacterial flora [21].

The microbes of *D. citri* were altered by many factors. It was reported that *Carsonella* and *Wolbachia* were the primary symbionts in different populations of *D. citri* [22]. The compositional shifts of microbes in different life stages of *D. citri* were analyzed, and a conclusion was obtained that the microbiota composition varied during the growth and development process [23]. Temperature and gender were confirmed as the other two significant parameters altering the bacterial communities of *D. citri* [24]. The effects of *C*Las on the bacterial communities of *D. citri* were also analyzed using 16 S rRNA amplification sequencing, and *Candidatus Profftella* showed a co-exclusion relationship with *C*Las [19]. Additionally, the gut microbial community of *D. citri* was also confirmed to be altered by different host plants [25].

After two weeks of feeding on two host plants, *M. exotica* and ‘Shatangju’ mandarin (*C. reticulate* Blanco cv. Shatangju), the adults were harvested, and the guts from various treatments were examined in this study. By using RNA-seq and 16 S rDNA amplification sequencing, comparative transcriptome and microbial community composition studies of gut samples from *D. citri* adults fed on various host plants were further examined. Our findings offer more fundamental knowledge to strengthen the bond between *D. citri* and host plants.

## Results

### Transcriptome sequencing, assembly, and annotation

After sequencing, more than 46 million raw and clean reads were yielded from each sample (Supplemental Table 2). A total of 41,795 unigenes were assembled by Trinity, with an average size of 986 bp. N50 length and GC content are 2177 bp and 41.51%, respectively. Only 23,154 unigenes, which accounted for 55.41% of all the unigenes, were annotated into at least one database, and the number of unigenes annotated in the Nr, KEGG, COG, and SwissProt databases was 20,812 (49.80%), 19,167 (45.86%), 14,741 (35.27%), and 16,625 (39.78%), respectively. All the raw reads have been submitted to the SRA database with the accession number PRJNA914050.

### DEG identification from different samples

**Fig. 1:**
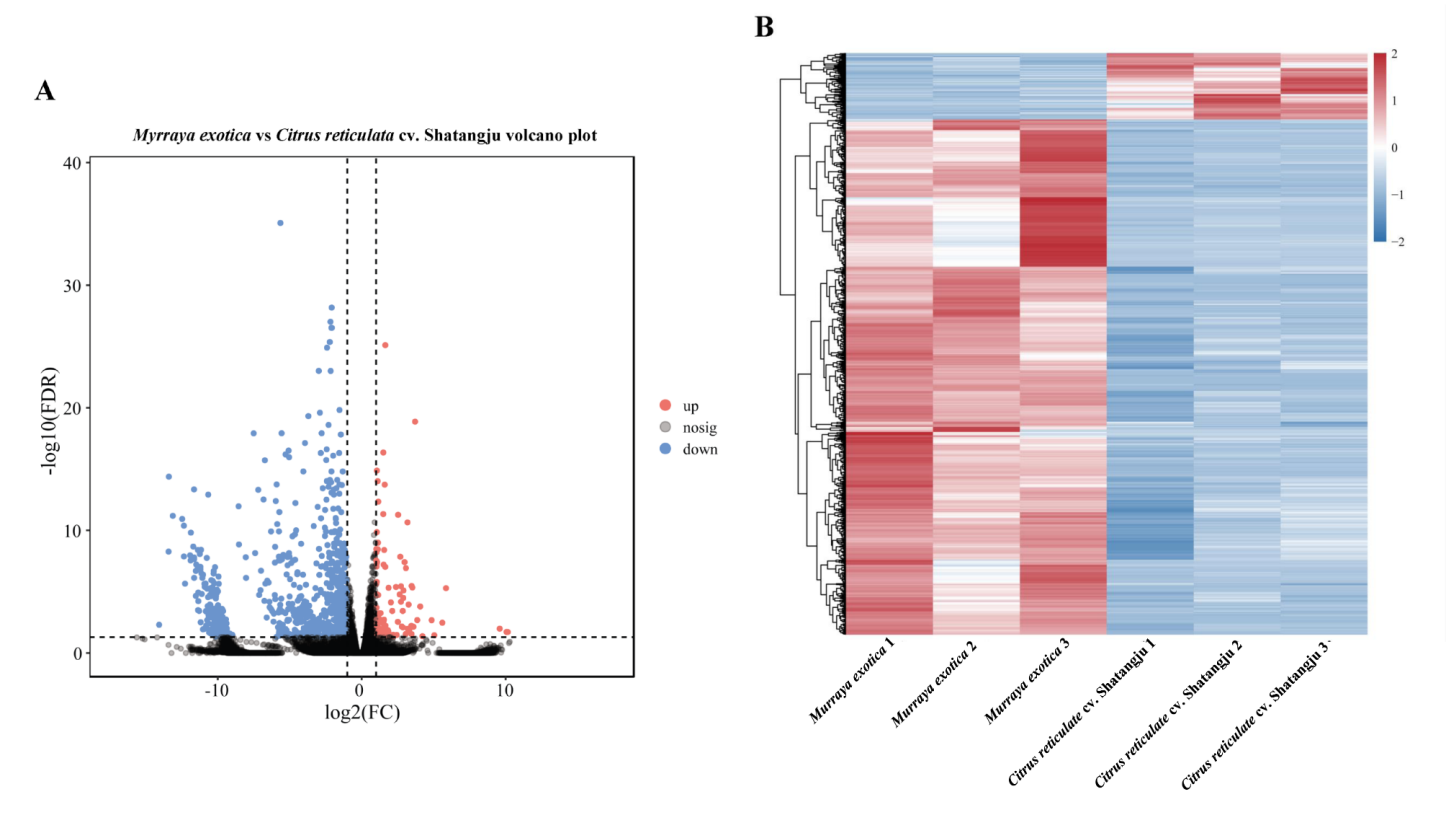
Differentially expressed genes (DEGs) found in the gut of *D. citri* adults reared on *M. exotica* and ‘Shatangju’ mandarin. **A**: The volcano plot of DEGs identified from the gut of *D. citri* adults reared on *M. exotica* and ‘Shatangju’ mandarin. The y-axis shows the value of − log_10_(FDR) of DEGs, and the x-axis shows the log_2_(Fold Change) of DEGs. Red and blue spots represented up- and down-regulated DEGs, while black spots indicated genes with no significant change in expression levels. **B**: Heatmaps of all DEGs. *Murraya exotica*: The gut samples obtained from the *D. citri* adults reared on *M. exotica*. *Citrus reticulate* cv. Shatangju: The gut samples dissected from the *D. citri* adults fed on ‘Shatangju’ mandarin. Heatmaps in red and blue indicated unigenes with higher and lower expression levels, respectively

**Fig. 2:**
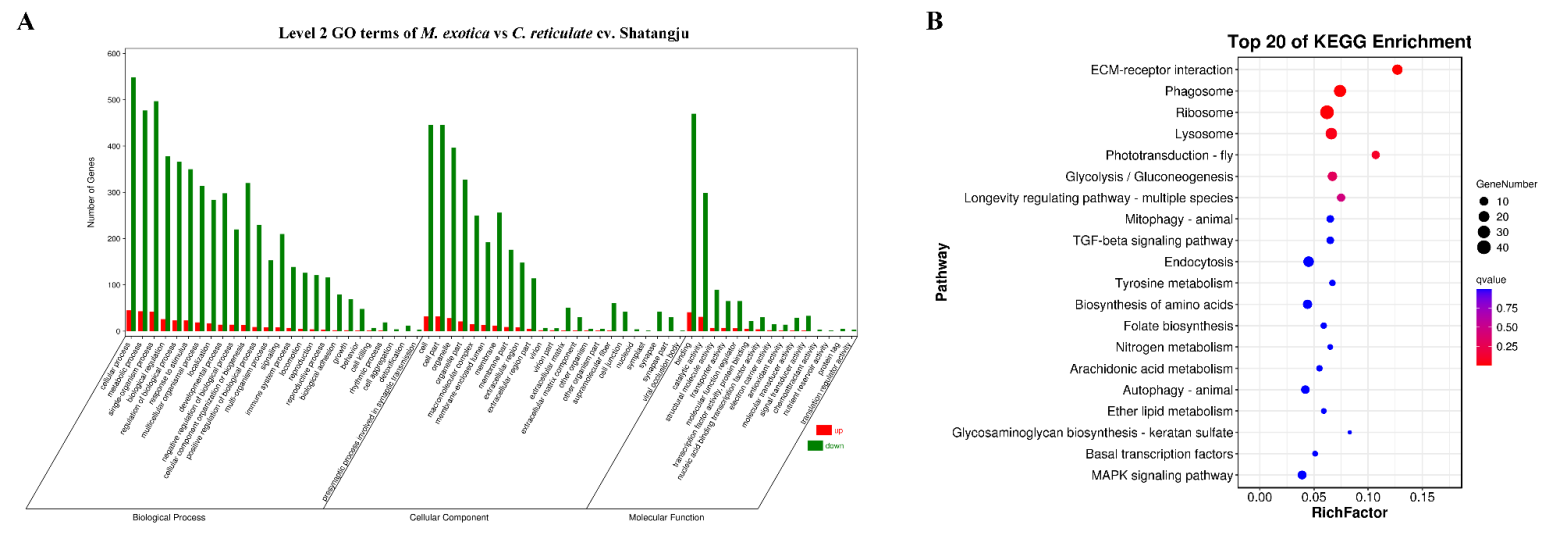
GO and KEGG enrichment analyses of DEGs. **A**: GO enrichment analysis of DEGs obtained from the comparative transcriptome analysis of gut samples with different treatments. The enrichment GO terms could be classified into three categories: biological process, cell composition, and molecular function. The up- and down-regulated DEGs were represented by red and green histograms, respectively. *M. exotica*: The gut samples obtained from the *D. citri* adults reared on *M. exotica*. *C. reticulate* cv. Shatangju: The gut samples dissected from the *D. citri* adults fed on ‘Shatangju’ mandarin. **B**: The top 20 KEGG pathways enriched with DEGs. Only seven pathways were significantly enriched with DEGs. The x-axis represents the rich factor

Based on the RNA-seq results, a total of 964 genes were associated with the different expressions in the gut of *D. citri* adults fed on *M. exotica* and ‘Shatangju’ mandarin. Compared to the group with *M. exotica* treatment, 110 DEGs displayed up-regulated expressions in the group treated with ‘Shatangju’ mandarin, whereas 854 DEGs were down-regulated (Fig. 1). For the GO enrichment analysis, the number of DEGs annotated in three categories, biological process, cellular component, and molecular function was 647, 528, and 674, respectively. The three most significantly enriched terms in these three categories were ‘cell motility’, ‘extracellular region’, and ‘macromolecular complex binding’, with 106, 156, and 127 DEGs annotated, respectively. The 402 DEGs enriched in KEGG pathways could be divided into five categories: metabolism, genetic information processing, environmental information processing, cellular processes, and organismal systems. Only seven pathways were significantly enriched, including ECM-receptor interaction (17 DEGs annotated, with p value = 3.83E-06), Phagosome (28 DEGs annotated, with p value = 1.30E-04), Ribosome (40 DEGs annotated, with p value = 2.53E-04), Lysosome (24 DEGs annotated, with p value = 1.82E-03), Phototransduction - fly (8 DEGs annotated, with p value = 4.53E-03), Glycolysis/Gluconeogenesis (13 DEGs annotated, with p value = 1.86E-02), and Longevity regulating pathway - multiple species (8 DEGs annotated, with p value = 0.032) (Fig. 2).

### Function analysis of DEGs

**Fig. 3:**
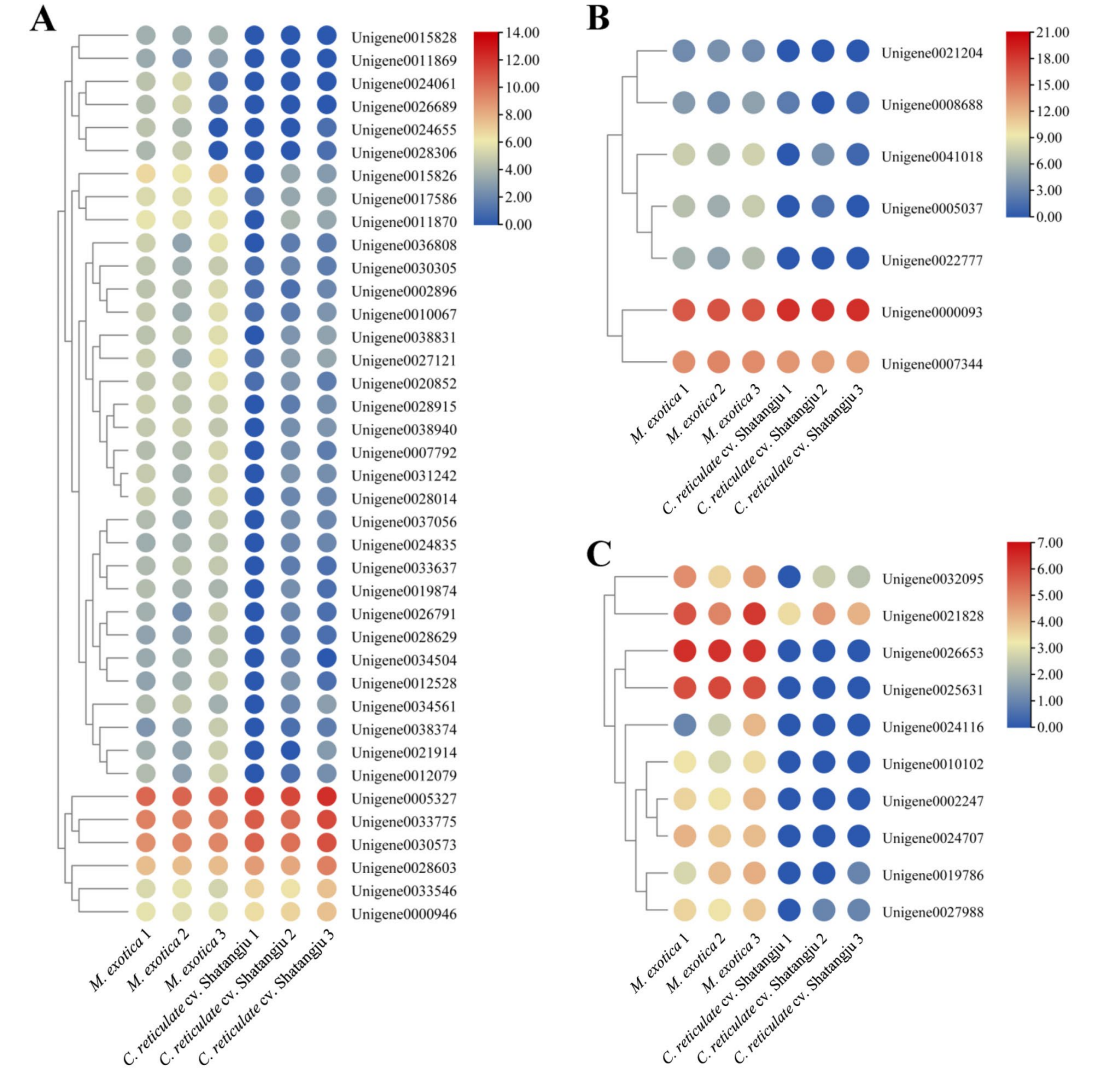
Heatmaps of selected DEGs identified from the comparative transcriptome analysis of gut samples with different treatments. **A**: Heatmaps of DEGs encoding ribosomal proteins. **B**: Heatmap of DEGs encoding cathepsins. **C**: Heatmap of DEGs involved in mitochondrial respiratory chain complexes. Heatmaps in red and blue indicated unigenes with higher and lower expression levels, respectively. *M. exotica*: The gut samples obtained from the *D. citri* adults reared on *M. exotica*. *C. reticulate* cv. Shatangju: The gut samples dissected from the *D. citri* adults fed on ‘Shatangju’ mandarin

According to the KEGG enrichment results, the ribosome pathway was significantly enriched. Therefore, the DEGs that encode ribosomal proteins were identified. As shown in Fig. 3, a total of 39 DEGs encoding ribosomal proteins were identified in the samples with ‘Shatangju’ mandarin treatment when compared to those in the samples treated with *M. exotica*, of which six were up-regulated and 33 were down-regulated. In addition, the seven DEGs encoding cathepsin, which have crucial functions in the lysosome, were also examined; all of them showed down-regulation in the samples after being treated with ‘Shatangju’ mandarin treatment, except for the gene encoding cathepsin B-like precursor (Unigene0000093). Moreover, a few genes connected to the mitochondrial respiratory chain were also discovered to be DEGs. When compared to the samples treated with *M. exotica*, the expression of two genes encoding NADH dehydrogenase, five expressing cytochrome c oxidase, and three encoding ATP synthase was down-regulated in the samples with ‘Shatangju’ mandarin treatment. Our findings suggest that *D. citri* adults can adapt to different host plants by regulating gut plasticity at the transcriptional level.

### Verification of DEGs by qRT-PCR

**Fig. 4:**
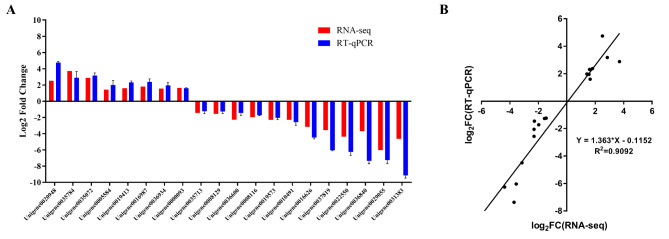
The results of quantitative real-time PCR (qRT-PCR) validation. **A**: qRT-PCR validation of 20 selected DEGs based on transcriptomic data. Among them, 8 DEGs were up-regulated and 12 DEGs were down-regulated in the group treated with ‘Shatangju’ mandarin when compared to the group with *M. exotica* treatment. *EF1α* and *RPS20* were used as references. **B**: Correlation analysis between transcriptome and qPCR validation results

To confirm the correctness of RNA-seq results, eight up-regulated genes and twelve down-regulated genes were chosen for qRT-PCR experiments. As shown in Fig. 4, the fold changes of DEGs calculated from the qRT-PCR data were fairly equivalent to the results of RNA-seq, with an R^2^ of 0.9092 to the fitting curve. These outcomes demonstrate the accuracy of RNA-seq data.

### The statistical analysis of the 16 S rDNA sequencing results

As shown in Supplemental Table 3, a total of 633,897 raw reads were obtained from the 16 S rDNA sequencing of six samples. After quality control, 626,672 effective tags for all samples, or at least 99,440 effective tags for each sample, were obtained. Cluster analysis found that the OTU counts in several samples ranged from 410 to 599. 1690 OTUs were identified from all the samples, including 31 phyla, 85 classes, 163 orders, 233 families, 354 genera, and 79 species. The OTUs in the samples treated with *M. exotica* were annotated into 30 phyla, 78 classes, 142 orders, 184 families, 247 genera, and 58 species. At the same time, the OTUs identified from the samples with ‘Shatangju’ mandarin treatment were grouped into 26 phyla, 67 classes, 137 orders, 194 families, 284 genera, and 63 species. The raw reads have been submitted to the SRA database with accession numbers from PRJNA847270.

### The gut microbial community composition is affected by host plants

**Fig. 5:**
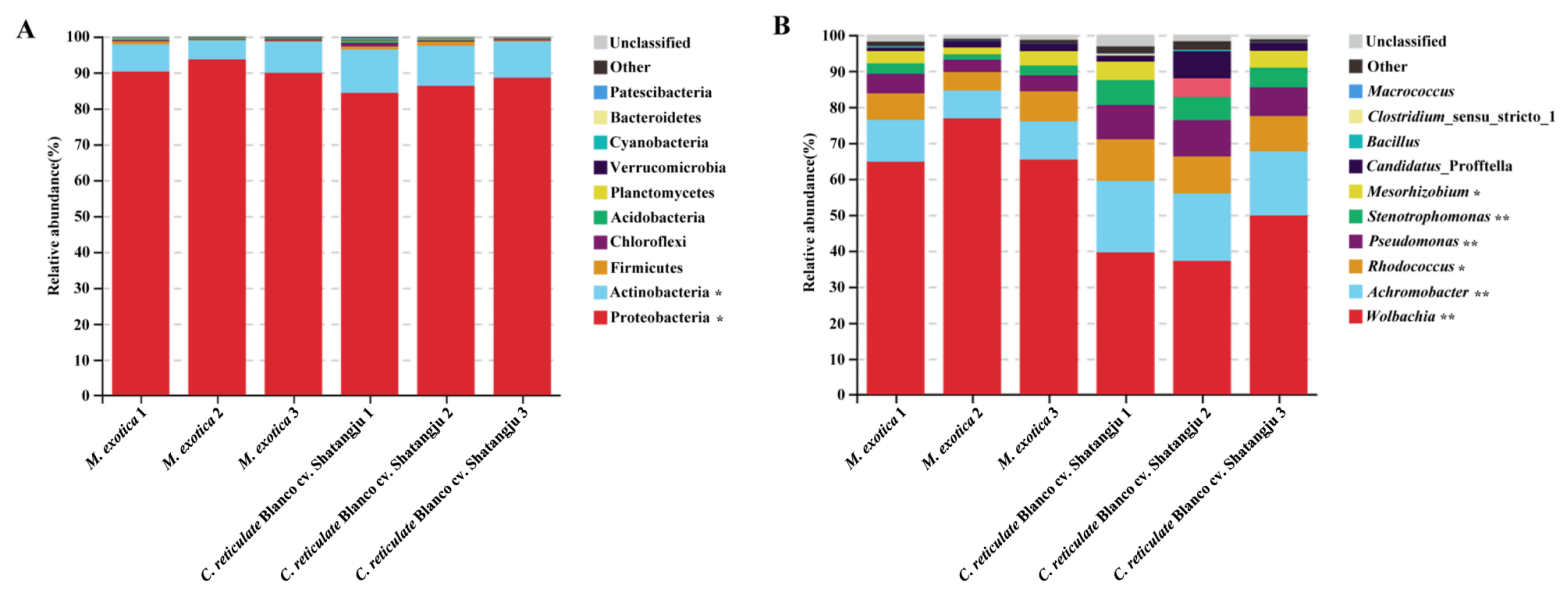
The gut microbial community composition in different groups. **A**: Relative abundances of the bacterial phylum in the gut samples of *D. citri* adults reared on different host plants. **B**: Relative abundances of the bacterial genus in the gut samples of *D. citri* adults reared on different host plants. *M. exotica*: The gut samples obtained from the *D. citri* adults reared on *M. exotica*. *C. reticulate* cv. Shatangju: The gut samples dissected from the *D. citri* adults fed on ‘Shatangju’ mandarin. * and ** indicate that the relative abundance of corresponding phyla and genera has significant differences in the comparison between these two groups (* *p* < 0.05; ** *p* < 0.01)

The composition of the gut microbial community in different groups was analyzed. As shown in Fig. 5, the phyla Proteobacteria and Actinobacteria were found to be the most abundant in all the samples. More than 84% of the gut microbes were Proteobacteria, demonstrating the significance of this phylum. Proteobacteria and Actinobacteria abundance varied significantly between samples from different host plant feedings (Fig. 5A). Besides, *Wolbachia*, *Achromobacter*, *Rhodococcus*, *Pseudomonas*, *Stenotrophomonas*, *Mesorhizobium*, and *Candidatus*_*Profftella* were the most abundant genera, which accounted for more than 1% of the gut microbes. Compared to the samples with *M. exotica* treatment, the abundance of *Achromobacter*, *Rhodococcus*, *Pseudomonas*, *Stenotrophomonas*, and *Mesorhizobium* was raised in the samples with ‘Shatangju’ mandarin treatment, while the amount of *Wolbachia* was decreased significantly (*P* < 0.05) (Fig. 5B). These data suggest that the composition of the gut microbial community in *D. citri* was affected by different host plants.

### Indicator species analysis in different groups

**Fig. 6:**
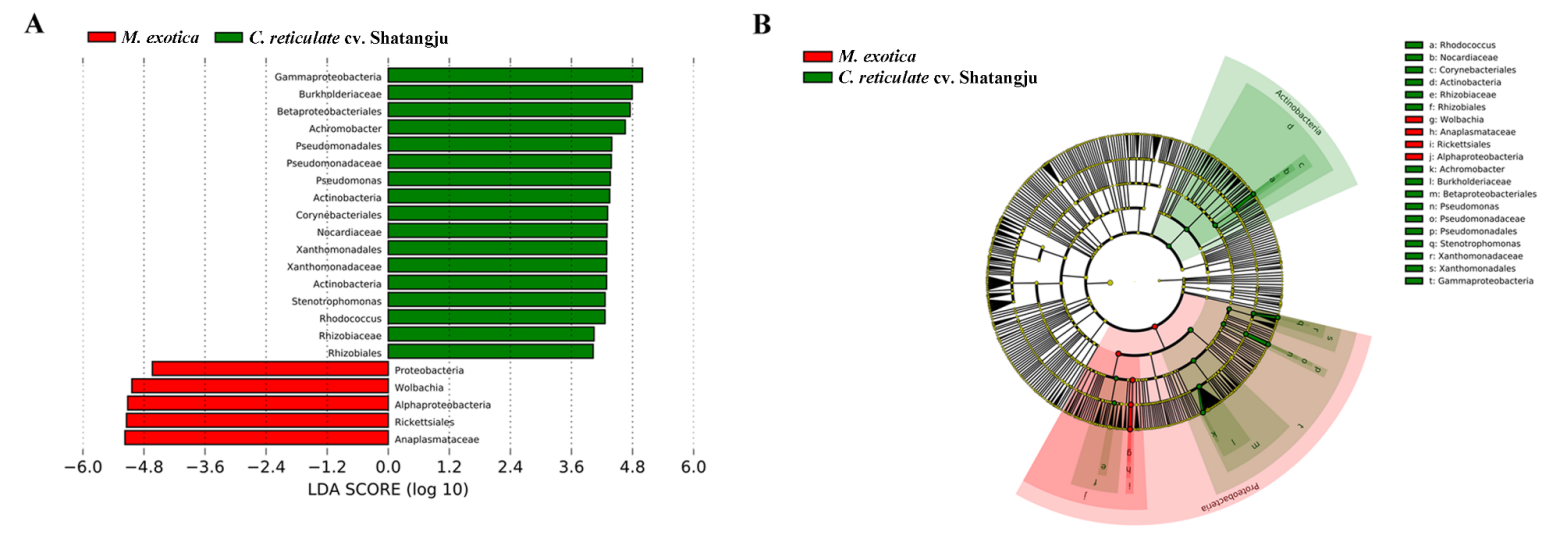
The indicator species found by LEfSe analysis. **A**: Bacterial taxa in the gut microbiota of *D. citri* feeding on different host plants with a linear discriminant analysis (LDA) score greater than two. **B**: Cladogram of bacterial biomarkers, from the phylum (innermost ring) to genus (outermost ring) level, with the standard of an LDA score > 2. Differential bacterial taxa in different groups are marked using lowercase letters. Each small circle at different taxonomic levels represents a taxon at that level, and the diameter of the circle is proportional to its relative abundance. The species with no significant difference were colored yellow, and the other different species as the group with the highest abundance of the species. *M. exotica*: The gut samples obtained from the *D. citri* adults reared on *M. exotica*. *C. reticulate* cv. Shatangju: The gut samples dissected from the *D. citri* adults fed on ‘Shatangju’ mandarin

To initially comprehend the differences in the species makeup between these two groups, the Venn analysis was carried out at the genus level. 71 genera were endemic to the group fed on *M. exotica*, as well as 112 genera in the group with ‘Shatangju’ mandarin treatment. Also, the indicator species in different groups were further investigated by LEfSe analysis. As shown in Fig. 6, the specific main flora in different groups was found. One genus, *Wolbachia*, was significantly enriched in the group fed on *M. exotica*. In addition, one family, Rhizobiaceae, one genus, *Stenotrophomonas*, three species, including *Rhodococcus erythropolis*, *Achromobacter xylosoxidans* subsp. xylosoxidans, and *Pseudomonas brenneri*, were more abundant in the groups with ‘Shatangju’ mandarin treatment. These microorganisms may serve as generic biomarkers for different samples.

### Microbial diversity in different groups

The alpha diversity indices showed that different host plants affected the gut microbial diversity. The results of the Shannon index value revealed that the diversity of gut microbiota in the group fed on ‘Shatangju’ mandarin was significantly higher than that of the group fed on *M. exotica* (Table 1). The group reared on ‘Shatangju’ Mandarin also had a greater Simpson index value, indicating that host plant changes altered the concentration degree of gut dominating bacteria. Moreover, there was no discernible difference in the Chao 1 and ACE indices, which suggests that there has been no significant change in the richness of *D. citri*’s gut microbiota.

**Table 1:** The alpha diversity indices of gut bacteria in *Diaphorina citri* adults reared on different host plants

index	sobs	shannon	simpson	chao	ace
*Murraya exotica* *Citrus reticulate* cv. Shatangju	547.33 ± 13.28 562 ± 34.04	2.702 ± 0.26 2.90 ± 0.53*	0.69 ± 0.05 0.73 ± 0.11*	614.39 ± 10.66 632.58 ± 34.90	623.06 ± 6.96 632.49 ± 17.36

Note: *Murraya exotica*: The gut samples obtained from the *D. citri* adults reared on *Murraya exotica*. *Citrus reticulate* cv. Shatangju: The gut samples dissected from the *D. citri* adults fed on *Citrus reticulate* cv. Shatangju

**Fig. 7:**
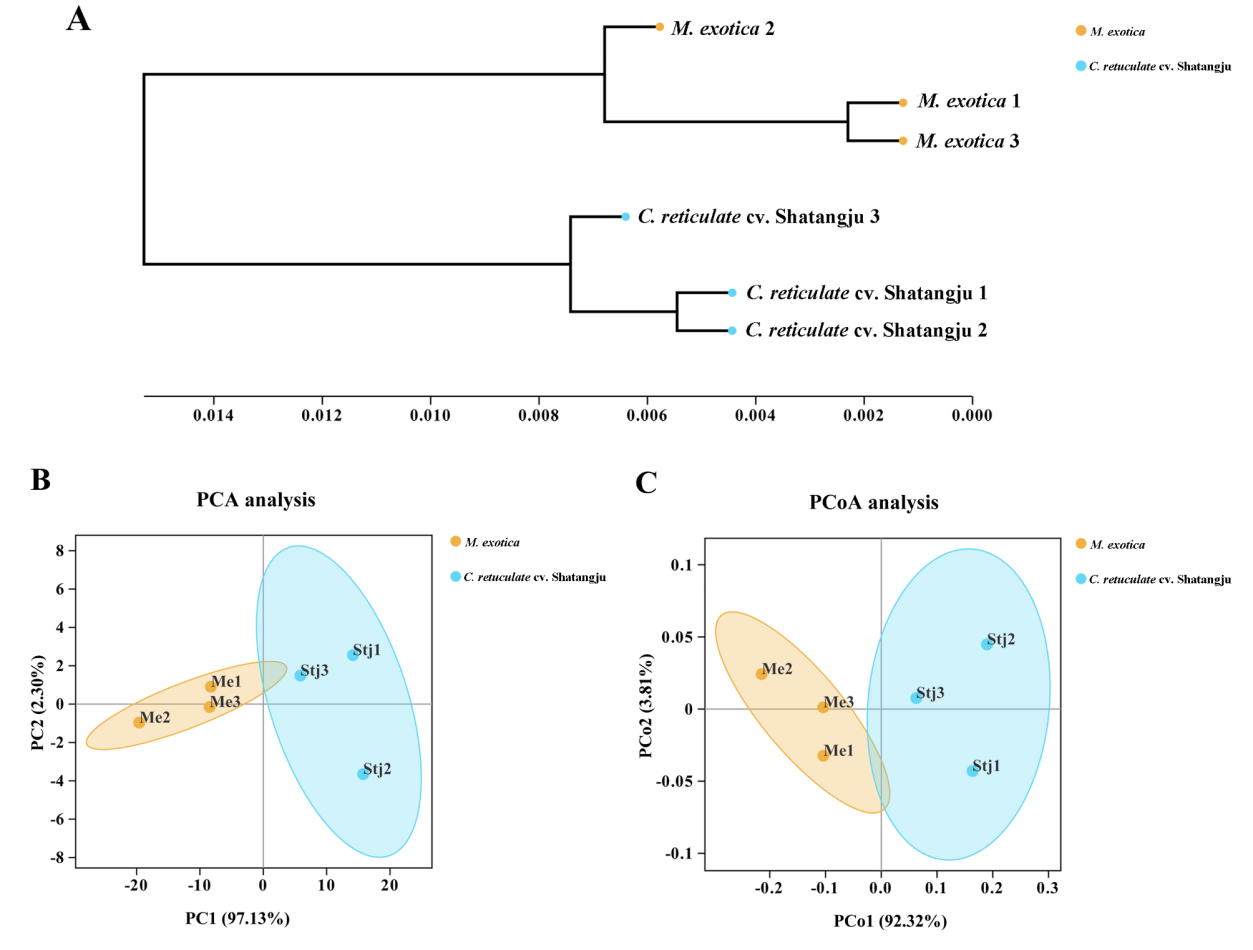
The Beta diversity analysis of the gut bacterial community of *D. citri* in different groups. **A**: A genus-level hierarchical clustering tree. **B**: PCA analysis on the OTU level in all samples using the weighted UniFrac method; **C**: PCA analysis on the OTU level in all samples using the weighted UniFrac method. Me and *M. exotica*: The gut samples obtained from the *D. citri* adults reared on *M. exotica*. Stj and *C. reticulate* cv. Shatangju: The gut samples dissected from the *D. citri* adults fed on ‘Shatangju’ mandarin

The beta diversity studies revealed that the gut microbial diversity of *D. citri* was altered by different host plants. As shown in Fig. 7A, the UPGMA cluster analysis at the genus level showed that different samples from the same group were grouped together, but two major divisions were seen between the different groups. The findings of PCA and PCoA produced using the weighted UniFrac method showed that two separate clusters exist among these samples, and the samples in different groups have a good degree of dispersion (Fig. 7B C).

### Functional prediction of gut bacteria

**Fig. 8:**
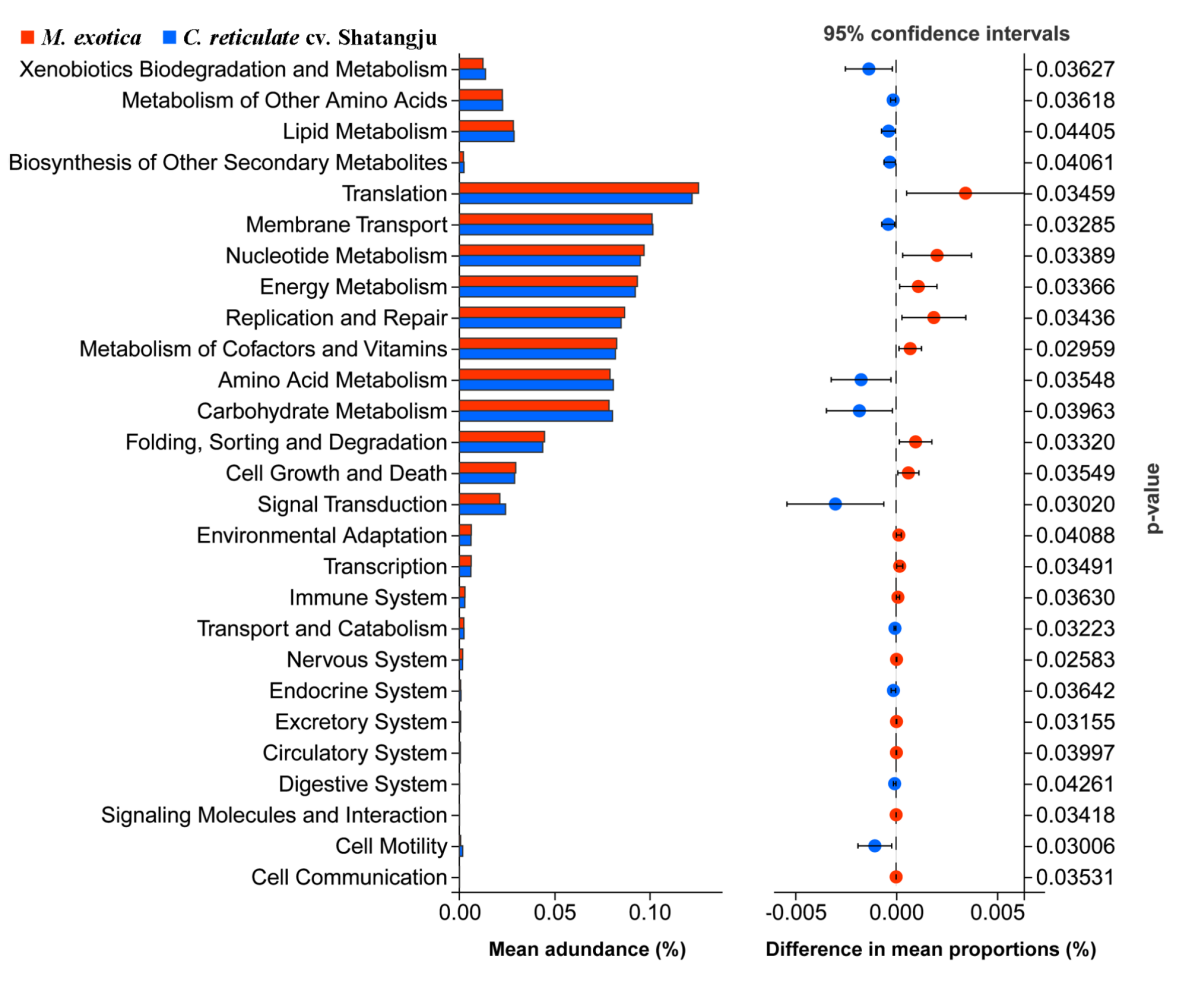
Comparison of predicted KEGG pathway functions in the gut bacteria associated with *D. citri* adults feeding on different host plants. The ordinate represents the KEGG pathway, which is significantly different in this comparison. The blue represents the *C. reticulate* cv. Shatangju samples, and the red represents the *M. exotica* samples. *M. exotica*: The gut samples obtained from the *D. citri* adults reared on *M. exotica*. *C. reticulate* cv. Shatangju: The gut samples dissected from the *D. citri* adults fed on ‘Shatangju’ mandarin

To understand the function of the gut bacteria of *D. citri* in response to different host plants, Tax4Fun software was used for functional prediction according to the sequencing data and KEGG database. As depicted in Fig. 8, a total of 27 KEGG pathways were significantly changed in the comparison between these two groups. According to the categorization by function, the significant genes for pathways included four pathways for cellular processes, three for environmental information processing, four for genetic information processing, nine for metabolism, and seven for organismal systems were enriched. Among these pathways, the genes implicated in fifteen pathways were found in the group reared on *M. exotica*, while others were more enriched in the group fed on ‘Shatangju’ mandarin. For the pathways in metabolism, the abundance of significant genes involved in three pathways, including nucleotide metabolism, energy metabolism, and metabolism of cofactors and vitamins, was higher in the group reared on *M. exotica*. While the genes assigned to other six pathways, including amino acid metabolism, carbohydrate metabolism, xenbiotics biodegradation and metabolism, lipid metabolism, and biosynthesis of other secondary metabolites, were more abundant in the group fed on ‘Shatangju’ mandarin.

### Integration analysis of RNA-seq and 16 S rDNA sequencing

It is commonly accepted that the host is affected by the gut bacterial community through interactions with the expression of specific genes in the host’s gut tissue. In this study, no DEG-encoding peptidoglycan recognition protein was found in the comparative transcriptomic analysis, but 17 DEGs annotated into the pathway of ECM-receptor interaction were identified, which could be responsible for the significantly changed microbial community. In addition, we also found that the amino acid metabolism pathway of the gut bacteria was significantly affected by different host plants, and the changes in the bacteria could lead to changes in the ribosome pathway in the gut at the transcriptional level. Therefore, we speculated that the reaction of *D. citri* to different host plants might be the result of the combined impacts of gut gene expression and the bacterial community.

## Discussion

As an important citrus pest, *D. citri* can feed on many species of the Rutaceae family. Insects moving between multiple hosts is a frequent occurrence in natural ecosystems [4]. Herbivorous insects obtain their nutrients mainly from the host plants, and different quality plants could alter the physiological indicators of insects, such as their survival rate, size, weight, and so on [5]. It was reported that *D. citri* thrived and developed practically identically on the ‘Shatangju’ mandarin and orange jasmine *M. exotica*, which were both considered the most suitable hosts. In three host plants, including *Bergera koenegii*, *M. exotica*, and *C. sinensis*, substantial differences in metabolites, such as amino acids and sugars, were identified in prior research, indicating the different components in different host plants [26]. Therefore, we speculate that *D. citri* adjusts some of its own functions in response to different hosts to ensure superior growth and development. In this study, we have studied the differences in the gut gene expression profiles and bacterial community in *D. citri* adults reared on two host plants, *M. exotica* and ‘Shatangju’ mandarin. These findings shed fresh light on the interaction between *D. citri* and its host plants. Our results indicate that this pest regulates gut gene expression and gut microorganisms in response to different host plants. In addition, it has been determined that host plants affect the gut gene expression profiles and bacterial community composition and diversity of numerous insects, such as *Anoplophora glabripennis*, *Leptidea sinapis*, *Altica fragariae*, and *Altica viridicyanea*, which was confirmed again with *D. citri* [27–29]. These data provide a theoretical foundation for understanding the host plant adaptation mechanisms of *D. citri* adults.

As the principal nutrition access system from the environment, the insect gut has the functions of supplying energy to the organism, maintaining homeostasis, and shielding the insect from hazardous microorganisms or plant defense substances [30]. Molecular-physiological adaptations of the insect gut in response to different host plants have also been investigated by RNA-seq [29, 31]. In this study, the comparative transcriptomes of *D. citri* adult guts in response to two distinct host plants, *M. exotica* and ‘Shatangju’ mandarin, were investigated. These results imply that diverse molecular adaptations of *D. citri* adult guts occurred in response to different host plants. Among the differentially expressed genes, a few detoxifying genes were found, indicating that the plant defense substances in the two host plants might not differ greatly.

KEGG enrichment results showed that the ribosome pathway was significantly different in different groups, and 39 ribosomal proteins with differential expression were found. Previous studies indicated that differentially expressed genes involved in protein biosynthesis are associated with increased plasticity, allowing for the utilization of a broader range of host plants [28, 32]. Moreover, differentially expressed ribosomal genes could mediate the effects of host plant ribosome-inactivating proteins [28]. Our findings further support the previous studies, and the gut’s differentially expressed ribosome genes may be important for the host adaptation of *D. citri* adults. The lysosome pathway was identified as another significantly enriched pathway with DEGs, and seven cathepsins with different expressions were found. For phloem sap-feeding insects, cathepsins are the major digestive enzymes that hydrolyze dietary proteins in gut proteolysis [33]. Therefore, it is plausible to hypothesize that variations in the protein composition of various host plants may be responsible for differences in the gene expression of these cathepsins in the gut of *D. citri* adults. Furthermore, the mitochondrial respiratory chain (complex I–V), as one of the elements of the electron transport chain (ETC), is responsible for the aerobic primary energy production in eukaryotic cells [34]. In this study, several genes encoding NADH dehydrogenases, cytochrome c oxidases, and ATP synthases, which are the essential parts of mitochondrial complexes I, IV, and V, had down-regulated expressions in the samples with ‘Shatangju’ mandarin treatment when compared to those in the samples treated with *M. exotica*. These results suggest that more ATP could be synthesized by the gut cells of *D. citri* adults fed on *M. exotica*. Our results offer a fresh perspective on the adaptability of *D. citri* adults to host plants at the molecular level.

Recently, the gut bacterial communities of *D. citri* altered by different host plants were reported [5]. In this study, we found that a short period of feeding time on different plants was sufficient to alter the gut microbes of *D. citri*, further supporting and adding to the result reached by Meng et al. [5]. In this study, the gut microbiome of *D. citri* was dominated by Proteobacteria, actinobacteria, and firmicutes, which is similar to that found in other hemipteran insects, such as *Pyrrhocoris apterus*, *B. tabaci*, *Laodelphax striatellus*, and *Adelphocoris suturalis* [35–38]. Proteobacteria were identified as the most dominant phylum in *D. citri*, and were responsible for degrading the plant secondary metabolites and participating in the host plant adaptation [24, 25, 39]. Our results showed that there were fewer proteobacteria present than in the previous studies conducted by Meng et al. [24] and Jiang et al. [25]. Different experimental materials can be to blame for the discrepancy. The gut tissues were dissected for our experiment, whereas the entire insects were used in the previous studies. Besides, a significant difference in proteobacteria abundance was found in the comparison between samples with *M. exotica* and ‘Shatangju’ mandarin treatments. These results indicate that the variations in plant phloem sap could lead to changes in the proportion of proteobacteria. By creating secondary metabolites with antibacterial effects against parasites, pathogens, or parasitoids, actinobacteria are frequently found as protective symbionts for host insects [40, 41]. Actinobacteria and a variety of host insects have a nutritional mutualism relationship, according to Salem et al. [41]. In this study, actinobacteria were the dominant phylum in *D. citri*, highlighting the importance of this phylum. Different host plants had a considerable impact on actinobacteria abundance, indicating that actinobacteria may be more sensitive to host plant adaptation. In addition, firmicutes, which were functional in nutrient absorption, energy metabolism, and metabolism of plant secondary metabolites, were found to be another dominant phylum in this study [42, 43]. Although having a low proportion of gut microorganisms, our findings further demonstrate the significance of this phylum in the host plant adaptation strategy of *D. citri*.

In addition, a significant difference in the abundance of *Wolbachia*, *Achromobacter*, *Rhodococcus*, *Pseudomonas*, *Stenotrophomonas*, and *Mesorhizobium* between these two groups was also identified. *Wolbachia* have been found to have a variety of effects on the biology of host insects, including manipulating the reproduction of host insects through cytoplasmic incompatibility induction, and behaving as defensive symbionts for host insects by guarding against different infectious organisms and preventing the transmission of insect-borne pathogens and parasites [44–46]. *Achromobacter* assists insects to decompose and utilize organic matter, and the species from the gut of *Periplaneta americana* showed antibacterial activity [47]. *Rhodococcus* was also found to have multiple functions, including producing B-complex vitamins for *Rhodnius prolixus* and exhibiting strong contact inhibitory efficacy against four phytopathogens in *Delia antiqua* [48, 49]. *Pseudomonas* degrades chemicals from host plants and exhibits antagonistic activity against entomopathogenic fungi [50, 51]. *Stenotrophomonas* has been shown to metabolize a wide range of organic substances and provide essential amino acids to the host insects [52, 53]. Uncertainty surrounds *Mesorhizobium*’s role in the digestive system of insects. The study’s variation in bacterial abundance could be attributed to the different composition of plant phloem. Given the action and functional diversity of the bacterial genera mentioned above, we speculate that these microorganisms play significant regulatory roles in the fitness of their insect hosts. The *D. citri* bacterial community analyses not only provide useful information for the mechanism of insect-host plant interaction, but also hold promise for applied research in pest control with new approaches to genetically engineering these gut microbes to reduce insect fitness [18].

Gut microbial diversity is determined by numerous factors, including life stage, environmental habitat, geography, sex, and host plants [18, 54, 55]. The findings of α and β diversity analyses further supported the assertion that the alterations in host plants change the gut microbial diversity of *D. citri* adults. However, ANOSIM analysis demonstrated no significant difference existed (ANOSIM, r = 1, *P* = 0.10). The difference in the results obtained by different analyses could be related to the similar compound composition of these two host plants in the Rutaceae family. In addition, LEfSe research revealed that these two groups only differed significantly in a small number of genera. Further research revealed the main indicators for the group treated with *M. exotica* and ‘Shatangju’ mandarin, respectively, to be the genera *Wolbachia* and *Stenotrophomonas*. These results further reveal the significance of *Wolbachia* and *Stenotrophomonas* for the gut microbiome of *D. citri* in response to different host plants.

Gut bacterial function classification suggested that several KEGG pathways appeared to have undergone significant alteration in samples with different host plants fed. Our results support the functional plasticity of the gut microbes of *D. citri* in response to different host plants. A previous study showed that the metabolites of the phloem sap, including amino acids and sugars, were different in the comparison between citrus and *M. exotica* [26]. Our results demonstrated that numerous metabolic pathways of *D. citri* were affected by different host plants, and different KEGG pathways were enriched in different groups. The enriched pathways in different groups may be linked to the composition of phloem sap, indicating the importance of functional plasticity in the gut microbiome for insect host plant adaptation.

## Conclusion

In conclusion, the differences in gut gene expression profiles and bacterial composition and diversity of *D. citri* adults in response to two host plants, *M. exotica* and ‘Shatangju’ mandarin, were assessed. Our results provide additional evidence that host plants affect gut gene expressions and the composition and diversity of the bacterial community of *D. citri*. Comparative transcriptome analysis found that the genes coding for ribosomal proteins, cathepsins, and mitochondrial respiratory chain complexes show varied expression patterns in different groups. Besides, microbiome analysis showed that the genera *Wolbachia* and *Stenotrophomonas* were shown to be the main indicators for the group with *M. exotica* and ‘Shatangju’ mandarin treatments, respectively. The functions of nine metabolic pathways were significantly enriched in different groups. Our study accurately represents variations in the gut gene expressions and microbial community composition of *D. citri* in response to different hosts. These results also revealed the plasticity of the gut of *D. citri* and a preliminary understanding of the host plant adaptation mechanism of *D. citri*.

## Methods

### Insects and plants

A colony of *D. citri* that originated from a field collection close to Guangzhou has been maintained in our laboratory with *M. exotica* without insecticide exposure for more than one year. This laboratory population fed on *M. exotica* was kept at 25 ± 1 °C, 70% relative humidity, and a 12:12 (L:D) h photoperiod. With permission, the plants, *M. exotica* and ‘Shatangju’ mandarin, were bought from Guangdong Tianhe Fine Seed Nursery Stock Co., Ltd.

### Treatment and sample collection

The three-day-old adults of *D. citri* were fed with three-year-old, well-growing plants of *M. exotica* and ‘Shatangju’ mandarin. The adults received two weeks of feeding before being surface-sterilized in a 75% ethanol solution for 15 s, and then three times washed in sterile water for 30 s. The adults were dissected in a sterilized phosphate-buffered saline (PBS) solution, and the whole gut tissues were washed three times in PBS. Finally, the tissues from different samples were stored in a sterile PBS solution at -80°C. A total of 200 whole gut tissues of *D. citri* were collected as one replicate, and each treatment was carried out with three biological replicates. As a control, samples taken from adults fed *M. exotica* were used; as a treatment group, samples taken from adults fed ‘Shatangju’ mandarin were used.

### RNA-seq and differentially expressed gene identification

Total RNA was extracted from gut samples using the TRIzol™ Reagent (Invitrogen, Carlsbad, CA, USA) according to the manufacturer’s instructions. The integrity of total RNA was evaluated with agarose gel electrophoresis and an Agilent 2100 bioanalyzer (CA, USA). The purity and concentration of total RNA were analyzed with a NanoDrop 2000 (Thermo Fisher, MA, USA) and a Qubit 2.0 Fluorometer (Life Technologies, CA, USA). The cDNA library construction was performed on the platform of Gene Denovo Biotechnology Co. (Guangzhou, China). The mRNA from different samples was enriched with Oligo (dT) magnetic beads and fragmented with the ultrasonic. The fragments were then reverse-transcribed with random primers and M-MuLV reverse transcriptase. Once RNAse H degraded RNA, second-strand cDNA was synthesized by DNA polymerase I. Double-stranded cDNA was purified, embellished with end repair, poly (A) addition, and ligated with Illumina sequencing adapters. The cDNA fragments were then amplified by DNA polymerase I once more after being purified using AMPure XP beads. Finally, the PCR products purified with AMPure XP beads were sequenced on the Illumina HiSeq4000 platform (Illumina, San Diego, CA, USA).

The raw reads were filtered, and the low-quality reads were removed with fastp software [56]. Trinity (http://trinityrnaseq.sourceforge.net/) was used to perform the clean read assembly. Then the assembled unigenes were annotated in NCBI protein nonredundant (NR), SwissProt, Kyoto Encyclopedia of Genes and Genomes (KEGG), and Clusters of Orthologous Groups of Proteins (COG) with an e value < 0.00001. Afterwards, the RSEM software (http://deweylab.biostat.wisc.edu/rsem/) was utilized for the quantitative analysis of the unigenes in different samples with the transcripts per million reads (TPM) approach. The differentially expressed genes (DEGs) in the comparison between two groups were identified with DESeq2 software with thresholds of FDR < 0.05 and |log_2_FC| > 1. Furthermore, GO and KEGG enrichment analyses were conducted, respectively.

### Validation of DEGs

Twenty DEGs identified from the comparison were employed for qRT-PCR validation. As shown in Supplemental Table 1, the primers for these DEGs were designed using Primer Premier 5 software and synthesized at Guangzhou Tianyi Huiyuan Gene Technology Co., Ltd. The cDNA synthesis was performed with 1.0 µg total RNA and a PrimeScript™ RT reagent Kit with gDNA Eraser (Perfect Real Time) (DRR047A, Takara, Dalian, China) according to the manufacturer’s instructions. The qRT-PCR process was carried out as we previously explained [57]. The genes elongation factor 1 alpha (*EF1α*) and ribosomal protein S20 (*RPS20*) were used as references, and the relative expression levels of DEGs in different samples were calculated using the 2^−ΔΔCT^ method.

### DNA extraction, PCR amplification, and 16 S rDNA sequencing

The genomic DNA of the gut samples was isolated using HiPure Soil DNA Kits (Magen, Guangzhou, China) following the operating instructions. The quality and concentration of each DNA sample were analyzed using NanoDrop 2000 (Thermo Fisher Scientific, USA). The integrity of the DNA sample was detected with agarose gel electrophoresis analysis.

The universal primers 341 F (5’-CCTACGGGNGGCWGCAG-3’) and 806R (5’- GGACTACHVGGGTATCTAAT-3’) were used for the V3-V4 region of 16 S rDNA amplification, and the extracted DNA was used as templates. The 50 µL PCR reaction mixture, which included 10 µL of 5 × Q5@ Reaction Buffer, 10 µL of 5 × Q5@ High GC Enhancer, 1.5 µL of 2.5 mM dNTPs, 1.5 µL of each primer (10 µM), 0.2 µL of Q5@ High Fidelity DNA Polymerase, and 50 ng of DNA sample, was carried out in triplicate. The following amplification procedures were employed: 95 °C for 5 min for one cycle, 95 °C for 1 min, 60 °C for 1 min, and 72 °C for 1 min for 30 cycles, followed by 72 °C for 7 min for one cycle. The PCR products were then purified using the AxyPrep DNA Gel Extraction Kit (Axygen Biosciences, Union City, CA, USA). After being quantified, the PCR products were sequenced on an Illumina platform (Illumina, San Diego, USA).

### Quality control, clustering, and annotation

The raw data obtained from the sequencing was further filtered using FASTP (version 0.18.0), where the reads that had more than 10% of unknown nucleotides (N) and fewer than 50% of bases with a quality (Q-value) > 20 were removed. The clean reads were spliced with FLASH (version 1.2.11) software based on the standard of a minimum overlap of 10 bp and mismatch error rates of 2%. High-quality clean tags were then obtained by removing the noisy sequences from the raw tags. The clean tags were clustered into operational taxonomic units (OTUs) with ≥ 97% similarity using the UPARSE (version 9.2.64) pipeline. The chimeric tags were removed using the UCHIME algorithm, and the effective tags were used for further analysis. The tag sequence with the greatest abundance was chosen as a representative sequence within each cluster. The representative OTU sequences were classified into organisms by a naive Bayesian model using an RDP classifier (version 2.2) based on the SILVA database (version 132) with a confidence threshold value of 0.8.

### Community composition and indicator species analyses

The species composition of each sample at each taxonomic level was analyzed. The unique and common species or OTUs in these two groups were examined with Venn analysis and an upset plot analysis by Welch’s t-test and Wilcoxon rank test. The indicator species in different groups were identified with LEfSe software (version 1.0).

### Microbial diversity and function prediction analyses

Four indices of alpha microbial diversity analysis, including Chao1, ACE, Shannon, and Simpson, were analyzed with the QIIME (version 1.9.1) software. The index value comparison between these two groups was calculated using the Welch’s t test. Each sample’s OTU rarefaction and rank abundance curves were also computed. As for the beta diversity analysis, the sequence alignment was first performed using Muscle (version 3.8.31) and a phylogenetic tree was constructed using FastTree (version 2.1). Then a weighted unifrac distance matrix was generated by the GuniFrac package (version 1.0). PCA (principal component analysis) and PCoA (principal coordinates analysis) of the weighted unifrac distances were performed in the R project Vegan package (version 2.5.3). The enrichment analysis of KEGG pathways of the OTUs was inferred using Tax4Fun (version 1.0). The functional difference between groups was assessed with the Welch’s t test. These bioinformatics analyses were carried out using Omicsmart (http://www.omicsmart.com).

## Electronic supplementary material

Below is the link to the electronic supplementary material.

Supplementary Material 1

## Data Availability

The raw reads of transcriptomes in this study have been deposited in the NCBI SRA database with the accession numbers of PRJNA914050 and PRJNA847270.

## Abbreviations

*C*Las: *Candidatus* Liberibacter asiaticus
COG: Cluster of Orthologous Groups of proteins
DEGs: Differentially expressed genes
DGGE: Denaturing gradient gel electrophoresis
EF1α: Elongation factor 1 alpha
ETC: Electron transport chain
FC: Fold change
GO: Gene Ontology
KEGG: Kyoto Encyclopedia of Genes and Genomes
NR: NCBI protein nonredundant
qRT-PCR: Quantitative real time polymerase chain reaction
RFLP: Restriction fragment length polymorphism
RNA-seq: RNA-sequencing
RPS20: Ribosomal protein S20.
TPM: Transcripts per million reads
