# Supplementary material for: The comparison of gut gene expression and bacterial community in Diaphorina citri (Hemiptera: Liviidae) adults fed on Murraya exotica and ‘Shatangju’ mandarin (Citrus reticulate cv. Shatangju)
Source: BMC Genomics. 2023 Jul 24;24:416. doi: 10.1186/s12864-023-09308-2 (PMC10364414; doi:10.1186/s12864-023-09308-2)
Supplement: Supplementary file 1 — Supplementary Material 1 [file 12864_2023_9308_MOESM1_ESM.docx]

Supplement Table 1. Primers used for RT-qPCR in the study

| Gene ID | Forward Primer (5'-3') | Reverse Primer (5'-3') |
| --- | --- | --- |
| *EF1α* | GTGCCTGATTGAAGCTCTCG | GCATCTCGACGGATTTCACC |
| *RPS20* | GGAGGATTAGTCGACCCACC | CATTCTGATTGGGCCCTTGG |
| Unigene0020948 | TTTGGTGAAGAAAAGATCGGTG | CCTTGGCTCCATAGGTAGACG |
| Unigene0035784 | CCCAACGCAGACCCTAACTA | GTATGGGTAAGCTGCACTGC |
| Unigene0035072 | CACTACTAACCACCCCACAGGA | CTCAACAGGTATCACAGGCTCAC |
| Unigene0005584 | TTGCCATCCCCTCTCTTCAC | TGTCAACTTCGAGTAAACGGA |
| Unigene0019413 | ATTATGGCGAGGGTAGGAGC | GGATGCAGTGCCTTTCCAAA |
| Unigene0016987 | GATGTCCTCGGAGAAATGGC | CAATGAGACGGAATCTGTAGCG |
| Unigene0036934 | TCCAGGCGAACCTTTGATTG | TGAACACGGAAAACGACCC |
| Unigene0000093 | CGGTTTGTCGGTATCGTTGG | TGATCCTCGTCTGATTCGTCC |
| Unigene0035713 | GTTGACTGCTGCTCATTGCTT | CCTCTTTATCTTCCATCGCCTTA |
| Unigene0008129 | GTTTGAATGTGGAGCGAAGC | CCAGTGTTCCTATGGGCGTA |
| Unigene0036600 | GGAAACAGACAACGGAGCG | ATGAACATTGAAGGCAGAGCA |
| Unigene0008116 | AATCATCGCCTTAGAATCGGTA | CAGAATAATAACATCGGGTGCA |
| Unigene0019573 | CAGGCAGATCAACCCATCAC | CATCAACAGTTTCCTTCCCTTC |
| Unigene0010491 | CTGGAGCACATCTCGGCATA | AACCAAGACCATTGAGGAAGC |
| Unigene0016626 | GCAAACTGCTCATCGTCTCAA | GCAACCACTTCCAATAACTCTGT |
| Unigene0037819 | ATACAAACGCTTTGATACTGACCG | AACATGGCATCCAGCCTGAC |
| Unigene0022550 | TCAGCACCTCCAACTATTCCC | CCAAACCTGTCACTTTCTACCCT |
| Unigene0036840 | CCTCTATGCCAACACAGTGC | CCTGCTTGCTGATCCACATC |
| Unigene0029055 | CAAGCCTTCCTTTCACCCAC | CACTGTTCCCGTGCATCAAA |
| Unigene0031383 | GGGCCTCTTACACCTACCTC | TTCAGGTTCTTCTCCAGGGC |

Supplement Table 2. Summary of the transcriptome data

| Sample | Raw reads | Clean reads | Q20 (%) | Q30 (%) | GC (%) |
| --- | --- | --- | --- | --- | --- |
| *M. exotica* 1 | 48,778,738 | 48,577,686 | 97.54% | 93.28% | 44.33% |
| *M. exotica* 2 | 51,909,316 | 51,710,982 | 97.59% | 93.39% | 43.56% |
| *M. exotica* 3 | 46,448,850 | 46,246,166 | 97.56% | 93.34% | 43.41% |
| *C. reticulate* cv. Shatangju 1 | 55,986,486 | 55,784,838 | 97.67% | 93.52% | 42.77% |
| *C. reticulate* cv. Shatangju 2 | 50,253,202 | 50,072,200 | 97.69% | 93.59% | 43.58% |
| *C. reticulate* cv. Shatangju 3 | 67,795,490 | 67,568,916 | 97.83% | 93.92% | 42.46% |

Note: *M. exotica*: The gut samples obtained from the *D. citri* adults reared on *M. exotica*. *C. reticulate* cv. Shatangju: The gut samples dissected from the *D. citri* adults fed on ‘Shatangju’ mandarin.

Supplement Table 3. The statistics of 16S rDNA sequencing data from different samples

| Sample Name | Raw Reads | Clean Reads | Raw Tags | Clean Tags | Chimera | Effective Tags | Effective Ratio (%) |
| --- | --- | --- | --- | --- | --- | --- | --- |
| *M. exotica* 1  *M. exotica* 2  *M. exotica* 3  *C. reticulate* cv. Shatangju 1  *C. reticulate* cv. Shatangju 2  *C. reticulate* cv. Shatangju 3 | 100,831  108,980  106,651  107,817  102,491  107,127 | 100,748  108,909  106,568  107,730  102,423  107,060 | 100,078  108,605  106,184  107,346  102,015  106,746 | 99,737  107,849  105,823  106,918  101,656  106,302 | 297  539  378  93  118  188 | 99440  107310  105445  106825  101538  106114 | 98.62  98.47  98.87  99.08  99.07  99.05 |

Note: *M. exotica*: The gut samples obtained from the *D. citri* adults reared on *M. exotica*. *C. reticulate* cv. Shatangju: The gut samples dissected from the *D. citri* adults fed on ‘Shatangju’ mandarin.
